# Evaluation of the influence of N-acetylcysteine and broccoli extract on systemic paraquat poisoning: Implications for biochemical, physiological, and histopathological parameters in rats 

**DOI:** 10.22038/IJBMS.2024.75258.16311

**Published:** 2024

**Authors:** Mahdieh Raeeszadeh, Sara Arvand, Danial Shojaee Moghadam, Loghman Akradi

**Affiliations:** 1 Department of Basic Sciences, Sanandaj Branch, Islamic Azad University, Sanandaj, Iran; 2 Graduate of Faculty of Veterinary Sciences, Sanandaj Branch, Islamic Azad University, Sanandaj, Iran; 3 Department of Pathobiology Sciences, Sanandaj Branch, Islamic Azad University, Sanandaj, Iran

**Keywords:** Apoptosis, Broccoli, N-acetyl cysteine, Oxidative stress, Paraquat

## Abstract

**Objective(s):**

Paraquat (PQ), a potent environmental herbicide, is recognized for inducing irreparable toxic damage to biological systems. This study aimed to evaluate the effectiveness of N-acetylcysteine (NAC) and broccoli extract, individually and in combination, in alleviating PQ poisoning in rats, leveraging the exceptional anti-oxidant, anti-inflammatory, and anti-apoptotic properties of broccoli.

**Materials and Methods:**

Seventy Wistar rats were categorized into seven groups: C (control, vehicle), PQ (paraquat at 40 mg/kg), BC (broccoli extract at 300 mg/kg), NC (N-acetylcysteine at the same dose of 300 mg/kg), and combined groups PQ+BC, PQ+NC, and NC+PQ+BC, all administered equivalent doses. After 42 days, blood samples were collected to evaluate liver and kidney parameters, proinflammatory biomarkers, caspase-3, and caspase-9. Lung tissues were excised, with one part preserved for hydroxyproline and oxidative stress parameter measurement and another sectioned and stained for histopathological analysis.

**Results:**

The PQ group exhibited the highest lung-to-body weight (LW/BW) ratio, while the PQ+BC+NC group demonstrated the lowest ratio. Results indicated an elevated lung hydroxyproline concentration and a significant reduction in anti-oxidant enzymes (catalase, glutathione peroxidase, superoxide dismutase, and total anti-oxidant capacity) (*P*<0.001). The PQ+BC group showed modified malondialdehyde levels, reaching a peak in the PQ group. Additionally, a significant decrease in tumor necrosis factor, interleukin-1, caspase-3, and caspase-9 was observed in the PQ+BC+NC group (*P*<0.01). Pulmonary edema, hyperemia, and severe hemorrhage observed in the PQ group were notably reduced in the PQ+BC+NC group.

**Conclusion:**

The combination of active compounds from broccoli and NAC demonstrated significant systemic and pulmonary effects in mitigating PQ-induced toxicity.

## Introduction

Paraquat (PQ), an extensively used organic heterocyclic herbicide chemically designated as N, N-dimethyl-4,4-bipyridinium dichloride, finds widespread application in agricultural settings owing to its cost-effectiveness and efficiency. Unfortunately, it inflicts severe harm upon humans and animals upon contact (1). 

The deleterious impact of PQ predominantly focuses on the brain, kidneys, and liver. Nevertheless, accidental or deliberate poisoning with PQ yields more severe repercussions in the pulmonary system. PQ exposure prominently induces acute lung injury marked by severe hypoxia, edema, and respiratory dysfunction (2). This condition can eventually advance to pulmonary fibrosis, resulting in the permanent loss of lung tissue (3). According to the available literature, the mortality rate linked with PQ poisoning is as elevated as 60–80%, largely due to the absence of effective treatments (4).

The molecular mechanisms underlying PQ toxicity involve interference with pulmonary oxidative stress regulation, inflammatory response, epigenetics, apoptosis, autophagy, and the progression of lung fibrosis (5, 6). Previous studies have shown that PQ induces apoptosis in RAW264.7 cells in a dose-dependent manner through a caspase-dependent mitochondrial pathway, resulting in increased levels of caspase-3 and caspase-9. Additionally, exposure of RAW264.7 cells to PQ elevates intracellular levels of reactive oxygen species (ROS) (7, 8).

Another mechanism of PQ toxicity is associated with generation of ROS and reactive nitrogen species (RNS) through redox cycle processes, inducing mitochondrial oxidative stress and potential cell death. These mechanisms are considered crucial in paraquat-induced lung injuries (2). Furthermore, high levels of mitochondrial DNA fragments have been detected in the bronchoalveolar lavage fluids of paraquat-exposed rats, triggering the innate immune system’s response, leading to lung inflammation, inhibiting lymphocyte proliferation induced by lipopolysaccharide (LPS), and reducing interferon (IFN)-γ production and monocyte phagocytosis (9).

According to the literature, depletion of glutathione (GSH) exacerbates paraquat-induced poisoning, while addition of GSH to type-II alveolar cells offers protection against PQ toxicity (10). 

N-acetyl cysteine (NAC) functions as the precursor to acetyl-L-cysteine, which, in turn, serves as the precursor to GSH. Acting as an electron acceptor, GSH protects against oxidative damage. Consequently, the intracellular biosynthesis of glutathione plays a critical role in maintaining reduction and oxidation (REDOX) potential (11).

Broccoli, a green plant belonging to the cabbage family (Brassicaceae), contains two types of phytochemicals contributing to defenses against oxidative stress: 1) Direct anti-oxidants, actively engaging in redox reactions and removing oxidation products. Key direct anti-oxidants found in broccoli include vitamin C, phenolic compounds, carotenoids, and vitamin E. 2) Indirect anti-oxidants, encompass a diverse range of chemical structures capable of inducing cellular protection (12).

However, limited reports exist regarding the anti-oxidative effects of broccoli extract against PQ toxicity in the liver, kidney, and lungs. Therefore, the primary objective of this study is to demonstrate the anti-oxidative efficacy of broccoli extract as a source of natural anti-oxidants, coupled with NAC, in countering paraquat-induced toxicity. This examination will encompass biochemical, serological, and molecular-pathological perspectives in rats.

## Materials and Methods

This interventional-experimental study involved 70 male Wistar rats with an average weight of 220±30 g. The rats were housed under standard conditions, maintaining a 12/12-hr light/darkness cycle, at the School of Veterinary Medicine’s animal facility. They received appropriate food and water throughout the study. The research strictly adhered to animal ethics rules and regulations and obtained approval from the Sanandaj University Ethics Committee (code IR.IAU.SDJ.REC.1401.082).

Following a one-week adaptation period, the rats were randomly assigned to seven groups, each comprising 10 rats, and were subjected to the following treatments:

C group: Received a carrier substance in the same amount as the experimental groups.

PQ group: Received PQ dichloride orally at a dose of 40 mg/kg body weight per day.

BC group: Received intraperitoneal administration of 300 mg/kg of broccoli extract.

NC group: Received intraperitoneal administration of 300 mg/kg of N-acetyl cysteine (NAC).

PQ+BC group: Received both PQ and broccoli extract.

PQ+NC group: Received both PQ and NAC.

PQ+BC+NC group: Received PQ, broccoli extract, and NAC in their drinking water (13, 14).

The experiment extended over a period of 42 days. Upon concluding the study, blood samples were obtained from the anesthetized animals to isolate serum for assessing liver enzymes, pro-inflammatory factors, and total serum capacity. Following this, the animals were euthanized, and their lungs were excised and weighed. The right lung was preserved in formalin for the preparation of tissue sections, while the left lung was immediately used to generate tissue extracts for the measurement of oxidative stress parameters, caspase-3 and caspase-9 levels, and hydroxyproline content.

***Measurement of hydroxyproline concentration in the lung***

For the assessment of hydroxyproline content in the lung, a 1% homogenate of lung tissue in 1% hydrochloric acid was prepared. The hydroxyproline concentration was determined using a colorimetry method developed by Brien’O and Edwards, utilizing Ehrlich’s reagent along with chloramine T and a hydroxyproline standard (15).

***Measurement of biochemical parameters for liver and kidney function***

For the quantification of liver function biomarkers, including serum alkaline phosphatase (ALP) and serum aspartate aminotransferase (AST), as well as kidney function indicators, namely urea and creatinine (Cr), a Pars Azmoon Kit and an autoanalyzer device were employed (16, 17). 

***Determination of pro-inflammatory biomarker concentrations***

To determine the concentrations of pro-inflammatory biomarkers (IL-6, TNF-α, and IFN-γ) in the serum sample, we employed kits from Shanghai BOYAO Biotechnology, China, following the manufacturer’s instructions (18, 19). 

***Measurement of caspase-3 and caspase-9 concentrations***

The concentrations of caspase-3 and caspase-9 in the serum samples were determined using ELISA Kits (ZB-10280C-R9648 and ZB-10280C-R1249) (20).

***Measurement of oxidative stress biomarkers***

For the measurement of oxidative stress parameters, including glutathione peroxidase (GPx), superoxide dismutase (SOD), and catalase (CAT), Zellbio commercial kits (Zellbio, Veltinerweg, Germany) were employed, utilizing the optical absorption method. GPx activity was determined by the oxidation of NADPH to NADP+ with an ELISA reader. SOD activity was assessed by measuring the amount of the sample catalyzing the decomposition of 1 μmole of O_2_− to H_2_O_2_- and O_2_- in 1 min. CAT enzyme activity was defined by the reduction of 1 μmol of H_2_O_2_ per minute at 240 nm. Serum total anti-oxidant capacity (TAC) values were determined using the FRAP method (21).

In the FRAP method, the increase in the absorption of the TPTZ-Chloride Fe ш complex by the anti-oxidants in the serum was measured using a spectrophotometer at 593 nm (22). 

We also quantified malondialdehyde (MDA), the final product of lipid peroxidation, in the lung by utilizing the thiobarbituric acid method and reading the optical absorption at a wavelength of 532 nm (23).

***Histopathological analysis of lung tissues***

We obtained tissue sections from fixed lung tissue and stained them with H&E. Subsequently, we conducted a comparative evaluation of different groups in microscopic fields. Lesions, including edema, hyperemia, hemorrhage, and atelectasis, were graded on a scale from zero to three (0=none, 1=mild, 2=moderate, and 3=severe).

***Broccoli hydroethanolic extract preparation method***

Upon receiving approval from the Herbarium Center of Agricultural Research Center, we dried and powdered broccoli plants. Subsequently, 100 g of the powder was mixed with 450 ml of 80% methanol. After 48 hr, the mixture was filtered and concentrated under vacuum using a rotary evaporator.

*Determining total phenol, total flavonoid, and IC*_50_ *contents in broccoli extract*

The total phenolic content was measured using the Folin-Ciocalteu method, and the results were expressed in mg/mg gallic acid per gram dry weight of the plant extract. Additionally, the flavonoid content was determined by the aluminum chloride method and expressed in mg quercetin/g dry weight of the extract. Furthermore, the free radical inhibitory impact was determined by applying the DPPH (2,2-diphenyl-1-picryl-hydrazine-hydrate) method (24). 

*Isolation and identification of sulforaphane composition using a GC-MS device*

The sample was extracted using methanol, followed by dehydration with anhydrous sodium sulfate, employing an Agilent 7890B GC System/5977A. A 1 µl volume of the sample was injected into the device. We utilized a GC Column HP-5MS (30 m, 0.25 mm, 0.25 u) and maintained a column temperature of 280 °C. Identification of peak formation and determination of its concentration in the extract were achieved using the standard sulforaphane peak method (Sigma-Aldrich Co.) (CAS# 4478-93-7; Catalog#s4441) (25).

***Data analysis***

The data were presented as Mean ± Standard Error of the Mean (SEM). The analysis was carried out using SPSS version 26. The normal distribution of the data was assessed through the Kolmogorov-Smirnov test. Following the confirmation of data normality, a one-way ANOVA was conducted, and if a significant difference was observed, Tukey’s *post-hoc* test was employed to compare means. In the case of non-parametric histopathological lung data, the Kruskal-Wallis test was applied for examination. Significance levels of *P*<0.001, 0.01, and 0.05 were considered.

## Results

***Comparison of body weights, lung-to-body weight ratio, and hydroxyproline concentration***

According to Table 1, no significant changes were observed in the initial weight of animals across different groups. However, substantial variations emerged in the final body weight values among these groups. Notably, the PQ+BC+NC group recorded the highest mean final weight (245.25±1.35 g), while the PQ group had the lowest. Furthermore, a significant difference was evident when comparing the PQ group with the other groups regarding final weight (*P*<0.001).

Table 1 illustrates noteworthy differences among the experimental groups concerning the lung-to-body weight (LW/BW) ratio. The highest and lowest values were observed in the PQ (1.28±0.03) and NC (0.46±0.02) groups, respectively. A significant difference was notably observed between the PQ group and the other groups concerning this index (*P*<0.01).

Moreover, the results from Table 1 reveal the hydroxyproline content in the lung tissues, indicating the highest and lowest levels in the PQ (6.87±0.24 mg/g tissue) and NC (1.02±0.49 mg/g tissue) groups, respectively. In comparison to the other groups, the hydroxyproline content in the PQ group samples was significantly higher (*P*<0.001). Meanwhile, there were no significant differences observed in the means of this parameter between NC, BC, and C groups.

***Liver and kidney parameters***

**Figure 1:**
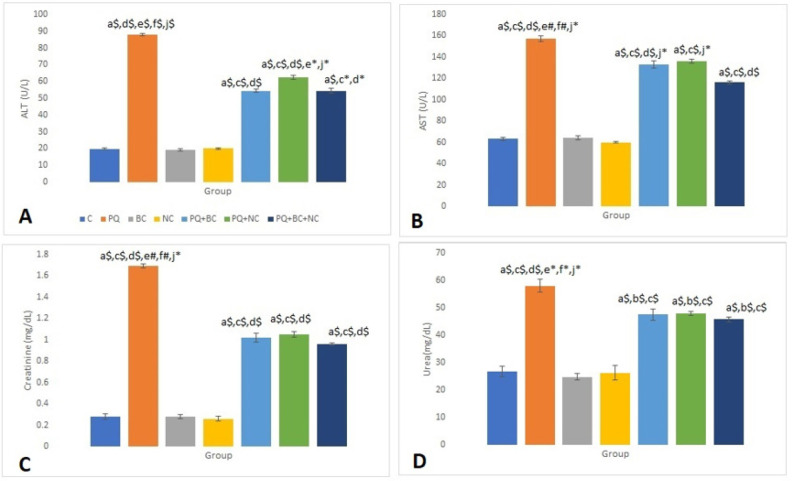
Evaluation of liver and kidney biomarkers among different study groups for 10 rats in each group based on one-way ANOVA and Tukey's *post-hoc *tests

Figure 1 presents the values of liver and kidney parameters across the various study groups. The highest and lowest ALT enzyme levels were observed in the PQ group (88±0.71 U/l) and BC group (19.25±0.72 U/l), respectively. A significant difference was found between the PQ group and the other groups regarding liver ALT levels (*P*<0.001). However, there was no significant difference between NC, BC, and C groups in this regard (Figure 1-C).

Aspartate transferase (AST) activity exhibited its highest and lowest levels in the PQ and NC groups, respectively. Along with the statistical difference between the PQ group and the other test groups regarding AST levels, there was a significant difference among all three PQ treatment groups (PQ+BC+NC, PQ+NC, and PQ+BC) when compared to the NC, BC, and C groups (Figure 1-D) (*P*<0.001 or *P*<0.05).

In this study, the highest and lowest mean creatinine levels were estimated at 1.69±0.02 mg/dl and 0.28±0.02 mg/dl, respectively, indicating the severity of kidney damage in the PQ group. Moreover, there was a significant difference between the PQ group and the other groups concerning creatinine levels (see Figure 1-E) (*P*<0.001 or *P*<0.01). Urea values across the different groups were reported as follows: C (26.75±1.91 mg/dl), PQ (58±2.37 mg/dl), BC (24.75±1.18 mg/dl), NC (26.25±2.56 mg/dl), PQ+BC (47.5±2.11 mg/dl), PQ+NC (46.75±0.72 mg/dl), and PQ+BC+NC (45.75±0.94 mg/dl). A significant difference existed between the PQ group and the other groups in this regard. Additionally, there was a significant difference between PQ+BC+NC, PQ+NC, and PQ+BC groups compared to C, PQ, BC, and NC groups concerning urea values (Figure 1-F) (*P*<0.001 or *P*<0.05).

***Changes in oxidative stress parameters***

Table 2 displays the values of oxidative stress indicators. PGx, functioning as an anti-oxidant enzyme, exhibited the highest activity levels in the BC group (3.30±0.14 µmol/mg protein) and the lowest in the PQ group (0.37±0.04 µmol/mg protein). A significant difference was noted when comparing these groups to the others in this context (*P*<0.001). SOD enzyme activity peaked in the BC group (86.25±1.42 U/mg protein), with the lowest activity observed in the PQ group. Simultaneously, the PQ+BC+NC combination groups demonstrated increased levels of this enzyme compared to the PQ+BC and PQ+NC groups.

Concerning CAT enzyme performance, the BC and NC groups exhibited the highest levels, while the lowest was detected in the PQ group. A significant difference was observed between the PQ+BC+NC and PQ+BC groups compared to the PQ+BC+NC and PQ+NC groups (*P*<0.001 or *P*<0.01).

In contrast to lung anti-oxidant enzymes, tissue malondialdehyde levels significantly increased in the PQ group, while they decreased in the BC group. These differences were highly significant between the groups (*P*<0.001).

TAC levels reached their peak (767.5±17.6 µmol/ml) in the BC group, whereas the lowest levels were observed in the PQ group. There was a significant difference between the BC group and the other test groups, except for the NC group (*P*<0.001).

***Results from pro-inflammatory biomarkers***

**Figure 2:**
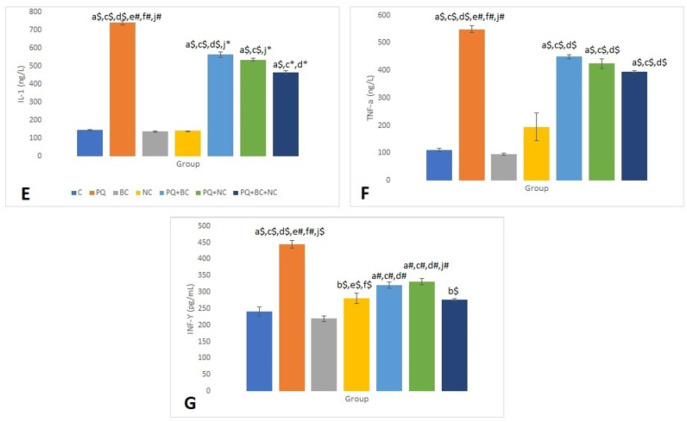
Comparison of differences in pro-inflammatory parameters (IL-1, TNF-α, and IFN- γ) among different study groups for every 10 rats in each group with one-way ANOVA and Tukey's post-hoc tests

Figure 2 illustrates the serum levels of IL-1 in different groups. The PQ and BC groups exhibited the highest and lowest IL-1 concentrations, respectively (136.25±4.1 ng/L). Furthermore, IL-1 levels in the PQ+BC, PQ+NC, and PQ+BC+NC groups decreased compared to the PQ group (562.5±14.72, 535±9.45, and 465±10.18 ng/L, respectively). These intergroup differences were statistically significant (*P*<0.01).

TNF-α concentrations reached their peak in the PQ group and were lowest in the BC group. Additionally, TNF-α levels decreased in the PQ treatment groups, with a significant difference observed between the PQ+BC+NC group and the C, NC, and BC groups (*P*<0.01).

The PQ group exhibited the highest IFN-γ level (447.5±11.14 pg/ml), while the BC and C groups showed the lowest levels (220±8.45 pg/ml and 242.5±13.72 pg/ml, respectively). A statistically significant difference existed between the PQ group and the other test groups (*P*<0.001). IFN-γ levels decreased in the PQ treatment groups, reaching the lowest level in the PQ+BC+NC group. Furthermore, a significant difference was noted between the PQ+NC and PQ+BC groups (*P*<0.01). No significant difference was observed between the PQ+BC+NC group and NC and C groups concerning the decrease in IFN-γ levels (Figure 2-I).

***Caspase-3 and caspase-9 levels***

The caspase-3 levels varied significantly among the groups, with the highest reported in the PQ group (15.32±0.27 ng/ml) and the lowest in the C group (9.32±0.19 ng/ml). Notably, a significant difference was observed between the PQ group and other groups in terms of caspase-3 levels (*P*<0.001). Moreover, all other treatment groups exhibited reduced caspase-3 levels, resulting in a significant distinction between PQ+BC+NC and C groups (*P*<0.001).

**Figure 3:**
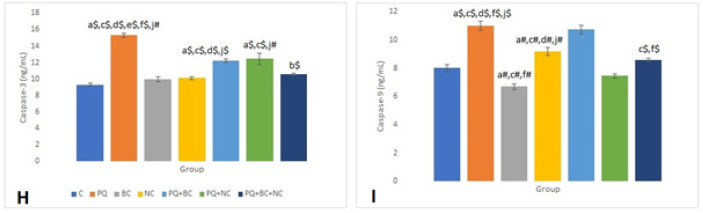
Comparison of caspase-3 and caspase-9 between groups. Mean ± SEM and *P*-values of 10 rats in each group was reported based on one-way ANOVA and Tukey's *post-hoc* tests

Similarly, the PQ group displayed the highest caspase-9 concentration (11.15±0.31 ng/ml), significantly differing from the other groups (*P*<0.001). Conversely, the BC group exhibited the lowest caspase-9 level (6.77±0.21 ng/ml), and this parameter decreased in the remaining PQ treatment groups (Figure 3).

***Histopathological evaluation of lung tissues***

**Figure 4:**
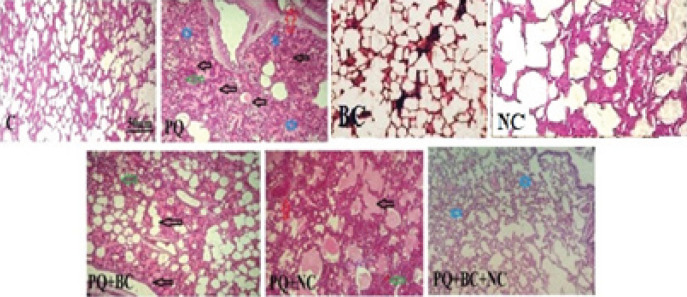
Histopathological examination of lung tissues in various groups of male rats (H&E staining, magnification ×400)

Histopathological assessment of lung tissues in various groups was performed, focusing on indicators such as edema, hyperemia, hemorrhage, and atelectasis (Table 3). The results indicate that the PQ group exhibits the most severe pathological damage, while the lowest degrees were observed in the other groups (Figure 4).

***Identification of active compounds in broccoli extrac*** **t**

 The analysis of Table 4 reveals the composition of compounds found in broccoli extract, showcasing the effective compounds and their respective concentrations. Specifically, sulforaphane, total phenol, and total flavonoids were identified in the broccoli extract.

**Table 1:** Comparison of body weights, lung-to-body weight ratio, and hydroxyproline concentration between groups of male rats Mean ± SEM and *P*-values of 10 rats in each group were reported based on one-way ANOVA and Tukey’s *post-hoc* tests

PQ+BC+NC	PQ+NC	PQ+BC	NC	BC	PQ	C	
210±2.93	208.25±2.58	208.75±2.53	209±2.75	207.5±2.11	210±2.52	208.75±2.79	Initial body weight (gr)
245.25±1.35^b$,e#,f*^	235.25±1.34^b$^	230.75±2.63^b$^	239.5±1.37^b$^	237.75±2.1^b$^	185±2.98	237.5±2.11^b$^	Final body weight (gr)
0.77±0.01 ^a^^*^^,b*, c*,d*^	0.86±0.03 ^a^^*^^,b#, c*,d*^	0.82±0.03 ^a^^*^^, b*, d*^	0.46±0.02 ^b#^	0.50±0.03 ^b#^	1.28±0.03 ^a#,c#,d#,e^^*^^,f^^*^^,j^^*^	0.59±0.02	Lung/ Body weight ratio
3.60±0.19^ a#,c$,d$, e#, f#^	5.47±0.07 ^a$,c$,d$,j#^	5.02±0.14^ a$,c$,d$,j#^	1.02±0.49 ^b$^	1.05±0.04 ^b$^	6.87±0.24 ^a$,c$,d$,e*,f*,j#^	1.20±0.07	Hydroxy proline concentration (mg/g tissue)

a: when compared to the control group, b: when compared to the PQ group, c: when compared to the BC group, d: when compared to the NC group, e: when compared to the PQ+BC group, f: when compared to the PQ+NC group, j: when compared to PQ+BC+NC group, ($ *P*<0.001, # *P*<0.01, * *P*<0.05)

A: Alanine transaminase (ALT; U/L), B: Aspartate transaminase (AST; U/L), C: Creatinine (mg/dl), and D: Urea (mg/dl); PQ animals received paraquat (200 mg/kg), BC animals received broccoli extract (300mg/kg), NC animals received N-acetylcysteine (300 mg/kg), PQ+BC animal received paraquat +broccoli extract,  PQ+NC animal received paraquat +N-acetylcysteine, PQ+BC+NC animal received paraquat +broccoli extract +N-acetylcysteine; a: when compared to the control group, c: when compared to the BC group, d: when compared to the NC group, e: when compared to the PQ+BC group, f: when compared to PQ+NC group, j: when compared to PQ+BC+NC group, ($ *P*<0.001, # *P*<0.01, * *P*<0.05)

**Table 2:** Comparison of stress biomarkers among groups was reported with Mean ± SEM and *P*-values for 10 rats in each group, utilizing one-way ANOVA and Tukey's *post-hoc* tests

PQ+BC+NC	PQ+NC	PQ+BC	NC	BC	PQ	C	
0.65±0.07 ^a$, c$, d$^	0.46±0.01 ^a$, c$,d$^	0.52±0.03 ^a$, c$, d$^	2.57±0.12 ^b$,e$,f$,j$^	3.30±0.14^ a*,b$,d#,e$,f$,j$^	0.37±0.04 ^c$,d$^	2.5±0.14 ^b$,e$,f$,j$^	GPx (µmol/mg protein)
37.25±1.04 ^a#,b*,c$, d$^	25±3.31 ^a#,b*, c$, d$^	27.25±1.44 ^a#,b*, c$, d$^	78.5±0.42 ^b$,e$,f$,j$^	86.25±1.42 ^b$, e$,f$,j$^	16.25±0.86 ^a$, c$, d$, e*, f*, j*^	71±1.58 ^b$,e$,f$,j$^	SOD (U/mg protein)
1.89±0.03 ^a*, b*, c#, d$^	0.82±0.01 ^a#,c$, d$, e#^	2.02±0.65 ^a#,b#,c#, d#, f$^	4.4±0.13 ^b$,e$,f$,j$^	4.77±0.22 ^a*,b$,e#,f$,j#^	0.82±0.05 ^a$,c$, d$, e$^	3.52±0.13 ^b,$,e#,f$,j$^	CAT (µmol/mg protein)
8.7±0.26 ^a$,b$, c$,d#^	6.95±0.26^ a$, b$,c$,d#^	7.0±0.26 ^a$,b$,c$,d#^	3.22±0.22 ^b$, d$, e#, f*, j#^	2.67±0.11 ^b$, d$, e$, f$, j$^	13.65±0.5 ^a$,c$,d$,e$,f$,j$^	3.45±0.13 ^b$,e$, f$, j$^	MDA (µmol/mg protein)
407.5±5.59 ^a$,b$,d$,e#, f#^	355±6.26 ^a$, b$, d$^	307.75±3.05 ^a$, b$, d$^	742.5±18.2 ^b$,e$,f$,j$^	767.5±17.6 ^b$,e$,f$,j$^	175±21.12 ^a$, c$, d$, e$, f$, j$^	642.5±16.5 ^b$,e$,f,$j$^	TAC (µmol/ml)

a: when compared to the control group, b: when compared to the PQ group, c: when compared to the BC group, d: when compared to the NC group, e: when compared to the PQ+BC group, f: when compared to the PQ+NC group, j: when compared to PQ+BC+NC group, ($ *P*<0.001, # *P*<0.01, * *P*<0.05)

E: interleukin (IL-1; ng/L), F: Tumor necrosis factor (TNF-α; ng/l), and G: Interferon (IFN- γ; ng/l); PQ animals received paraquat (200 mg/kg), BC animals received broccoli extract (300mg/kg), NC animals received N-acetylcysteine (300 mg/kg), PQ+BC animal received paraquat +broccoli extract,  PQ+NC animal received paraquat +N-acetylcysteine, PQ+BC+NC animal received paraquat +broccoli extract +N-acetylcysteine; a: when compared to the control group, c: when compared to the BC group, d: when compared to the NC group, e: when compared to the PQ+BC group, f: when compared to PQ+NC group, j: when compared to PQ+BC+NC group, ($ *P*<0.001, # *P*<0.01, * *P*<0.05)

**Table 3:** Histopathological evaluation in lung tissue among different groups with the Kruskal-Wallis test

PQ+BC+NC	PQ+NC	PQ+BC	NC	BC	PQ	C	
1.50 (1.00-1.70) ^a#,c*^	1.50 (1.20-1.50) ^a#,c*^	1.50 (1.00-2.00) ^a#,c*^	0.00 (0.00-0.50) ^b$^	0.50 (0.00-0.60) ^b$^	3.00 (2.5-3.00) ^a$^	0.00 (0.00-0.50)	Edema
1.00 (1.00-1.20) ^a#,c*^	1.60 (1.50- 2.00) ^a#,c#^	1.50 (1.00-1.50) ^a#,c*^	0.00 (0.00- 0.10) ^b$^	0.00 (0.00-0.00) ^b$^	2.00 (2.00-2.30) ^a$^	0.00 (0.00-0.00)	Hemorrhage
0.50 (0.50-0.60)	1.00 (0.70-1.00) ^a#,c*^	1.40 (1.00-1.50) ^a#,c*^	0.20 (0.00-0.50) ^b$^	0.00 (0.00-0.30) ^b$^	1.90 (1.10-2.00) ^a$^	0.00 (0.00-0.00)	Hyperemia
0.60 (0.50-0.80)	0.50 (0.50-0.60)	0.50 (0.40-0.70)	0.10 (0.00-0.50) ^b$^	0.00 (0.00-0.50) ^b$^	2.10 (2.00-2.50) ^a$^	0.10 (0.00-0.50)	Atelectasis

a: when compared to the control group, c: when compared to the BC group, d: when compared to the NC group

($ *P*<0.001, # *P*<0.01, * *P*<0.05)

Black arrow: edema, green arrow: hemorrhage, red arrow: hyperemia and blue star indicate atelectasis. PQ animals received paraquat (200 mg/kg), BC animals received broccoli extract (300mg/kg), NC animals received N-acetylcysteine (300 mg/kg), PQ+BC animals received paraquat +broccoli extract, PQ+NC animals received paraquat +N-acetylcysteine, PQ+BC+NC animal received paraquat +broccoli extract +N-acetylcysteine

**Table 4:** Total phenol, flavonoid, IC_50_, and sulforaphane concentrations in the broccoli extract

**%Sulphorphan**	**IC50**	**Total flavonoid** **(mg Q/g dw)**	**Total phenol** **(mg GAE/g dw)**	
**14.86**	19.76±0.15	40.1±3.16	52.40±4.51	Methanolic broccoli extract

## Discussion

PQ, a swift and cost-effective herbicide, presents a substantial risk of irreversible toxicity to both humans and animals. Consequently, this research aimed to evaluate the impact of broccoli extract and N-acetylcysteine (NAC) in alleviating the adverse effects of PQ on various body organs.

***Changes in final weight and the ratio index of lung weight to body weight in animals***

According to the findings of this study, there was a notable decrease in the final weight of animals in the paraquat (PQ) group, indicative of reduced food consumption and metabolic rates. This finding is consistent with previous studies (1, 26-28). However, the lost weight was restored after treating rats with broccoli extract and NAC, suggesting a positive impact on physical conditions, food consumption, and body weight. Another significant finding was the increased proline concentration in lung tissue, a pivotal organ during PQ treatment, which led to an increase in lung weight and subsequently elevated the lung-to-body weight (LW/BW) ratio in the PQ group. Treatment resulted in a reduction of this ratio.

***Alterations in serum liver and kidney enzymes***

Elevated levels of liver enzymes and biomarkers of kidney injury in PQ-induced poisoning indicated hepatocyte and nephron injury, attributed to oxidative stress and inflammation induced by the toxin. A significant rise in ALT, AST, and ALP, coupled with a notable increase in total creatinine and urea levels, was observed in the Paraquat-exposed group compared to the control group. These findings confirm the hepatotoxic and nephrotoxic potential of PQ. They align with previous studies investigating Paraquat-induced liver and kidney toxicity, which consistently reported an increase in serum levels and biochemical parameters.

Flavonoid compounds, such as those found in broccoli, are known for their physiological and biological roles, including anti-tumor, anti-oxidant, anti-diabetic, antiviral, and immune system-strengthening activities (29). Flavonoids have garnered extensive attention due to their multifaceted benefits, including anti-tumor, anti-oxidant, anti-diabetic, antiviral, and immune system-enhancing properties (30-33).

Previous research highlighted the protective effects of flavonoids in mitigating PQ-induced liver damage. For example, the flavonoid compound 8-Formylophiopogonanone B was considered protective against PQ-induced liver damage, while another study demonstrated the effectiveness of neohesperidin dihydrochalcone, a flavonoid-like compound, in mitigating PQ-induced liver damage through its anti-oxidant and anti-inflammatory effects (34, 35). Kidneys are also affected by PQ-induced poisoning, leading to increased serum creatinine levels, associated with nephron cell vacuolation and tubular necrosis (36, 37).

***Assessment of parameters related to oxidative stress***

Extensive research in this field indicates that the systematic inflammatory response syndrome (SIRS) may serve as a central pathogenic mechanism in PQ poisoning. PQ induces the generation of ROS within the body, leading to organ damage mediated by macrophages (38, 39). Macrophages, integral components of the innate immune system, play a crucial role in resolving inflammation and resisting invasion (40). PQ not only disrupts the oxidative cycle but also impedes the anti-oxidant system, thereby intensifying oxidative stress and inducing cellular damage either directly or indirectly (41). Consequently, the decrease in CAT, GPx, and TAC, coupled with an increase in MDA, signifies oxidative damage in paraquat-induced toxicity. In line with the findings of a recent study, the therapeutic effect of *Crocus sativus *(*C. sativus*) in healing lung lesions caused by PQ, along with dexamethasone exhibiting anti-oxidant and anti-inflammatory effects, is highlighted (42). Furthermore, the study confirms that systemic inflammation and oxidative stress induced by inhaled PQ were ameliorated with the use of chlorine, copper, and pioglitazone (Pio). Additionally, a synergistic effect between Pio, chlorine, and copper was demonstrated, suggesting the mediated effects of plant peroxisome proliferator-activated receptor gamma (PPARγ) and copper derivatives (43).

***Changes in pro-inflammatory biomarkers in the serum***

The invasion of PQ triggers a significant expression of pro-inflammatory mediator genes, leading to organ dysfunction and failure, thereby increasing mortality (44, 45). Inflammatory agents, such as IL-1 and TNF-α, are secreted during an inflammatory response by activated immune cells like lymphocytes and macrophages, amplifying inflammatory injury by promoting neutrophil prevalence and oxygen-free radical production (46). This escalation intensifies damage to body organs and tissues, potentially leading to apoptosis and cachexia. TNF-α, an inflammatory factor secreted by endothelial cells and mononuclear macrophages, activates nuclear factor-κB (NF-κB), mediating the expression of various inflammatory factors, promoting neutrophil degranulation, lysosome release, and exacerbating damage (47, 48).

Previous studies have demonstrated the beneficial effects of broccoli extract in inflammatory and oxidative stress conditions, including lead and arsenic poisoning and sperm cryopreservation (49, 50). Additionally, sulforaphane, found in broccoli, has been shown to protect damaged lungs from PM 2.5 exposure by reducing activation of the Nrf2/Keap1 pathway, thereby decreasing oxidative stress and inflammation (51). The reduction of pro-inflammatory markers IL-1, TNF-a, and IFN-γ observed during broccoli administration highlights the anti-inflammatory effects of this plant in paraquat-induced inflammatory damage. According to a recent study, *Curcuma longa* and curcumin were found to be effective in alleviating systemic and lung inflammation as well as oxidative stress caused by PQ, with curcumin demonstrating a more pronounced effect. In line with this study, the potency of broccoli was deemed even stronger (52).

To further validate these findings, another therapeutic investigation involving Z. multiflora and carvacrol demonstrated the improvement of unique markers associated with inhaled PQ. This underscores the anti-oxidant and anti-inflammatory potential of the medicinal plant and its compounds, particularly carvacrol, in addressing systemic biomarkers resulting from inhaled PQ (53). 

***Changes in caspase-3 and 9***

Caspase-3 plays a pivotal role in apoptosis by cleaving and activating caspases-6, -7, and -9, leading to the breakdown of apoptotic cells before their removal. Temporarily, caspase-3 activity is lost in the presence of thiol oxidizing agents such as diamide, oxidized glutathione, and dithiocarbamate disulfide. Therefore, broccoli extract and NAC have the potential to reduce caspase-3 levels, alongside oxidized thiol and glutathione groups. In our current research, caspase-3 and caspase-9 levels in animal serum increased following PQ-induced poisoning and decreased after treatment with broccoli extract and NAC. This aligns with findings by Gangyu Long *et al*., who reported that PQ-induced poisoning regulated the expression of VDAC and caspase in lung tissue, inducing acute lung injury (54).

***Histopathological evaluation of lung tissue in animals***

The histopathological assessment of lung tissues uncovered the highest levels of edema, hyperemia, and hemorrhage in the PQ group, which diminished in the PQ+BC+NC group. NAC and sulforaphane in broccoli extract collaboratively reduced hydroxyproline synthesis, delaying pulmonary fibrosis and mitigating lung lesions (55). These histopathological findings align with the outcomes of PQ toxicity, indicating a reduction in damage during treatment with NAC and broccoli, consistent with the biochemical results. In support of this observation, a study notes the early and late damage caused by PQ inhalation, suggesting that antibiotic compounds may have an impact in mitigating such damage (56). 

***Active compounds in broccoli extract and their effectiveness***

Anti-oxidant compounds have been implicated in mitigating PQ-induced damage. For instance, vitamin C, a potent free radical scavenger, is believed to reduce pro-inflammatory molecules in lung tissue and mitigate PQ-induced morphological changes in the liver (48). Vitamin E, acting as a ferroptosis inhibitor, plays a role in protecting against PQ-induced cellular damage. NAC, a precursor of glutathione, possesses anti-oxidant properties and can reduce the infiltration of inflammatory cells during PQ-induced poisoning (57). Consequently, the unique anti-oxidant properties of broccoli, which include vitamins C and E, as well as sulforaphane, demonstrate their significant ability to reduce oxidative stress and inflammation induced by PQ toxicity (58, 59).

## Conclusion

The study’s findings indicate that the combined application of broccoli and NAC is more effective in mitigating systemic damage induced by PQ compared to each substance individually. The synergistic therapy involving broccoli extract and NAC resulted in elevated levels of enzymatic anti-oxidants and Total Anti-oxidant Capacity (TAC), concurrently reducing IL-1, TNF-α, IFN-γ, and caspase-3 levels in paraquat-induced lung damage in rats. This reduction also extended to proline levels and the lung/body weight ratio, corroborating the observed pathological changes. Sulforaphane, present in broccoli extract and recognized for its anti-oxidant and antiproliferative properties, likely contributes to the amelioration of PQ poisoning. It is recommended that future research incorporates molecular investigations into gene expression to delve deeper into these mechanisms.

## Authors’ Contributions

M R and L A helped with conceptualization, data curation, formal analysis, investigation, methodology, and project administration. M R provided software support, supervision, validation, and visualization. M R, S A, and D SM wrote the original draft. M R and L A reviewed and edited the manuscript.

## Data Availability

The datasets used and/or analyzed during the current study are available from the corresponding author upon reasonable request.

## Funding Statment

There was no funding support for this article. 

## Conflicts of Interest

The authors declare no conflicts of interest.
